# Protocol of a randomized controlled trial of sun protection interventions for operating engineers

**DOI:** 10.1186/1471-2458-13-273

**Published:** 2013-03-26

**Authors:** Sonia A Duffy, David L Ronis, Andrea H Waltje, Seung Hee Choi

**Affiliations:** 1 School of Nursing, Department of Otolaryngology and Psychiatry, University of Michigan, Ann Arbor VA Center for Clinical Management Research, 400 North Ingalls Building #3178, Ann Arbor, MI, 48109-5482, USA; 2 University of Michigan School of Nursing, 400 North Ingalls Building #4330, Ann Arbor, MI, 48109-5482, USA; 3 University of Michigan School of Nursing, 400 North Ingalls Building #3217, Ann Arbor, MI, 48109-5482, USA; 4 University of Michigan School of Nursing, 400 North Ingalls Building #3219, Ann Arbor, MI, 48109-5482, USA

**Keywords:** Sun protection intervention, Skin cancer prevention, Sunscreen, Text messages, Worksite intervention

## Abstract

**Background:**

Skin cancer are increasing and some types of skin cancer are among the most lethal cancers yet are easily preventable. However, sun protection interventions are rarely implemented among outdoor workers. Our prior work shows that Michigan Operating Engineers (heavy equipment operators) spend an average of 4–5 hours in the sun, about one-third reported getting sun burned at least once a summer, and over half burned more than once a summer. About three-quarters of the sample never or only sometimes used sun block.

**Methods/design:**

Using the Health Belief Model as a guide, this randomized controlled trial (RCT) will test the efficacy of four sun protection interventions targeting Operating Engineers: a) education only; b) education and mailed sunscreen; c) education and text message reminders; and, d) education, mailed sunscreen, and text message reminders. Participations in the study will be offered during regularly scheduled safety trainings at the Local 324 Training Center. Pre- and post-intervention surveys will be collected to determine changes in sunscreen use and sun burning, the primary dependent variables. The analyses will include: a) paired t-tests to determine changes over time (from pre-intervention to post–intervention) in outcome variables (sunscreen use and burning) separately in the 4 intervention groups, b) Repeated Measures Analysis of Variance (RM-ANOVA) to compare the changes in outcomes across the 4 groups, and c) t-tests on change scores as follow-ups to the RM-ANOVA to determine exactly which groups differ from each other.

**Discussion:**

Based on the outcome of this study, we will develop a RO1 for wider scale testing and dissemination in conjunction with the International Training Center which services North America (including the US, Mexico, and Canada). Wide scale dissemination of an efficacious sun protection intervention has the potential to substantially impact skin cancer rates among this population. The ultimate goal is for high reach, high efficacy, and low cost.

**Trial registration:**

NCT01804595

## Background

The incidence of non-melanoma skin cancer is increasing [1,2] with the rates of melanoma increasing 3.1% annually since 1992 among non-Hispanic Whites [3]. While less common among Hispanics and Blacks, the rates of skin cancer are also increasing for Hispanics [4] and survival rates for Blacks are lower [5]. The risk factors predisposing a person to skin cancer include skin type, increased sun exposure, propensity to sunburns, sun burning and blistering throughout life, number of moles, and genetic susceptibility [3,6,7]. Outdoor workers are exposed to high ultra violet (UV) levels [7-9] increasing their risk of myeloid leukemia, malignant melanoma, and lip cancer [10]. Nonetheless, the rates of receiving skin examination and the use of sun protection are lower among outdoor workers compared to indoor workers [11,12], and only less than half of outdoor workers appropriately used sunscreen [13].

Previous studies have acknowledged several barriers to using sunscreen. A common belief is that those with tanned or olive skin are not at risk for skin cancer, thus protective measures need not be taken [14]. Positive attitudes towards tans are associated with decreased use of sunscreen, thus preventing outdoor workers from taking sun protection seriously [15]. Putting on sunscreen [16,17], is viewed as a hassle, and long sleeves are uncomfortable in the heat [15,18]. Men, which constitute majority of outdoor workers, may feel that it is not masculine to protect themselves from the sun [19], especially when around other males, while women feel that a tan makes them look slender and sexy [20]. In general, the perceived importance of sun protection is low among outdoor workers [21].

Operating Engineers, one group of outdoor workers, are responsible for the operation and maintenance of heavy earthmoving equipment used in the construction of buildings, bridges, roads, and other facilities [22]. Our prliminary data show that Operating Engineers are at greater risk of skin cancer since they spend an average of 4–5 hours in the sun, over 80% reported getting sunburned at least once per summer, and over half burned more than once a summer [23]. However, about three-quarters of the sample never or only sometimes used sun block and 23% showed interest in sun protection guidance [23].

The data support the need for sun protection interventions among this Operating Engineers. Therefore, this funded Blue Cross Blue Shield of Michigan RCT will determine the efficacy of four sun protection interventions—education only, education and mailed sunscreen, education and text message reminders, and education, mailed sunscreen, and text message reminders—among Michigan Operating Engineers. The specific aim is to determine differences in changes in sunscreen use and sun burning among Operating Engineers randomized to four sun protection interventions: a) education only; b) education and mailed sunscreen; c) education and text message reminders; and d) education, mailed sunscreen, and text message reminders.

### Theoretical framework

The Health Belief Model [24] was used to guide the development of the trial for Operating Engineers. The model proposes that behavior is influenced by four constructs including Perceived susceptibility (individual’s assessment of their risk of getting sunburned and subsequent skin cancer), Perceived severity (individual’s assessment of the seriousness of sun burning and subsequent skin cancer), Perceived benefits (individual’s assessment of the positive consequences of using sun protection), and Perceived barriers (individual’s assessment of the influences that facilitate or discourage adoption of sun protection behavior). The Health Belief Model also asserts that there are mediating factors including self-efficacy (confidence) [25], cues to action, socio-psychological variables, health motivation, and demographic variables. Among the four interventions, the educational component is designed to increase perceived susceptibility to and severity of sun burning and enforce the benefits of sunscreen use. Mailed sunscreen is designed to reduce barriers and will be served as a cue to action. Text messages are designed to emphasize perceived benefits and will be served as cues to action.

## Methods

### Design

**Figure 1:**
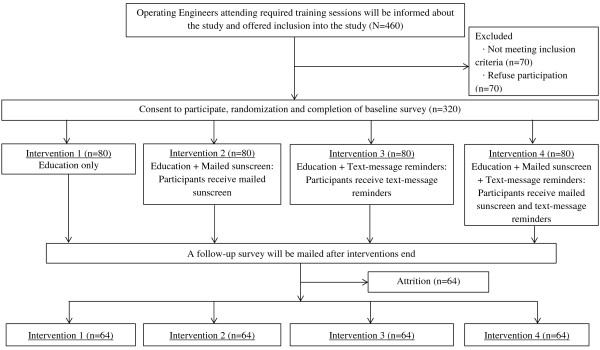
Experimental design overview.

This study will be a randomized controlled trial testing the efficacy of four sun protection interventions (Figure 1). Pre- and post-intervention surveys will be collected to determine the primary dependent variables (changes in sunscreen use and sun burning). Institutional Review Board approval has been obtained from the University of Michiga (HUM00057711).

### Setting/sample/power analysis

#### Setting and sample

One of the greatest strengths of this proposal is the “buy in” that we have from leadership at the Local 324 Training Center of the International Union of Operating Engineers. While leadership has always been interested in the health of their workers, this “buy in” is even greater now that the trend in Michigan is to shift health care costs to unions [26,27]. While the educational intervention given to all Operating Engineers will be provided during winter training sessions when Operating Engineers are typically not working, the text messages and mailed sun screen summer interventions will occur during the summer when UV rays are at their highest.

Inclusion criteria are Operating Engineers who: 1) are greater than 18 years of age; 2) are interested in enrolling in the sun protection study; 3) own a cell-phone that accepts text messages; and 4) are willing to share their phone number with the study team.

#### Power analysis

Though limited research has been conducted on interventions to increase use of sunscreen among outdoor workers, a medium sized effect (as defined by Cohen) [28] is plausible based on the studies by Armstrong et al., Buller et al., and Stock et al. [29-31]. Power analysis conducted with PASS software [32] indicated a need for 256 subjects to have 80% power to detect medium sized effects on changes in sunscreen use and sunburn in tests comparing the four individual treatment groups both by tests for mean scores with alpha of .05 two tailed. This sample size will provide even more power for tests of the effects of the interventions over time (pre- versus post- intervention) separately in the four groups.

Based on our prior experience with this group, we expect to approach 460 Operating Engineers of which we expect 15% (n = 70) to be ineligible (e.g., no text messaging or cell phone) and 15% (n = 70) to refuse, allowing for 320 to be consented. Since some analyses will be conducted just on subjects with complete data, this sample size was adjusted to account for a 20% attrition rate found in our previous research on this population resulting in a final sample size of 256. This can easily be done as there are 16,000 Operating Engineers in Local 324 all of which will be eligible and our prior work has estimated that about 23% (3,680) are interested in sun protection interventions [23]. Note, however, that the main analysis will include all subjects following an intent-to-treat approach so will have a larger sample size and thus slightly higher power.

### Procedures

#### Recruitment, randomization and Pre-intervention survey

Subjects will be recruited while they attend regularly scheduled safety training sessions provided by the Operating Engineers Local 324 Training Center. As has been done in our prior studies, the instructor or research nurse will briefly describe the opportunity to participate in research to the class. Those interested will be provided with an information pack which includes: a) an introductory information letter; b) Informed consent form; and c) the pre-intervention survey. The surveys will have random numbers on them from 1 to 4 assigning subjects to one of the four conditions. Both the study personnel and the subjects will be blinded to the condition of randomization. As requested by leadership at Michigan Local 324, all Operating Engineers, whether they enroll in the study or not, will receive the educational intervention during regularly scheduled training sessions conducted over the winter. Only those that enroll will be randomized, have their data collected, and receive the subsequent interventions during following June, July, and August.

#### Follow-up

In order to assess the primary outcomes—how often they used sunscreen and how many times they got sunburns in the prior month—subjects will be mailed a post-intervention survey in the fall. Subjects will receive $10 for each pre- and post-intervention survey.

### Description of sun protection interventions

#### Education only

Since studies have shown that increased knowledge regarding sunscreen use is efficacious in increasing awareness and fostering preventive health behaviors [33-36], a 30 minute power point presentation will be offered to Operating Engineers during their annual safety trainings. The content of the 30-minute didactic power point presentation was gathered from various sources including published articles, guidelines from the U.S. Food and Drug Administration (USFDA), the U.S. Preventive Services Task Force (USPSTF), and the American Academy of Dermatology (AAD). The content will include background information on the current use of sun protection among Local 324 members taken from a previous survey of this population [23], information on incidence and prevalence of skin cancer especially among outdoor workers, and the types of skin cancers and skin cancer risk. The content will also include methods to prevent sun burning including choosing from different products and reading Sun Protection Factor (SPF) labels recommended by the USFDA (2012) and USPSTF (2012), truths and myths about SPF [37], correct application of sunscreen, and other sun protection behaviors such as wearing hats, sunglasses, using shade, etc.

Adopting or tailoring information to address the needs of a specific population is important in order to overcome the perceived susceptibility, perceived severity, perceived barriers and increase the perceived benefits [29,36,38-41]. To make the presentation personal to Operating Engineers, pictures of Operating Engineers working in the sun will be scattered throughout. Visualization, such as pictures and graphs, enhances the understanding by taking the information to a more concrete level compared to verbal symbols alone [42]. Since the literature showed that pictures of skin cancer can motivate people to action [33] and inducing negative emotions can persuade people to act particularly when a solution is offered [25], pictures of skin cancer will be shown. Pictures on different SPF labels and sunscreens will be shown as well.

The second half of the session will be used for open discussion with Operating Engineers to address their perceived barriers and opinions about using sunscreen. This will allow for a greater understanding of the specific issues related to sun protection in the target population. By addressing the barriers to sun protection use and identifying strategies to overcome them, the participants are more likely to practice the desired behaviors [38-40].

#### Education and sunscreen

To reduce barriers to obtaining sunscreen and serve as cues to action, in addition to education, this group will be mailed sun screens three times over the summer, a supply of SPF30 sunscreen lotion (known to prevent sun burning and skin cancer) [43]. The mailing will consist of large bottles of sunscreen and a small bottle that can be refilled and attached to their huge key rings that hang off of their belts.

#### Education and text-message reminders

Recall of sun protection messages has been shown to be problematic [44], but the provision of text-message reminders has been found to increase adherence to sunscreen application in one study [31]. Hence, in addition to education and as cues to action, this group will receive 60 unique cellular telephone text-messages in the morning on three random days per week for the months of May, June, July, August and September. Using an internet text-messaging service, the messages will be computer generated and sent to Operating Engineers and contain information about weather conditions and various reminders (e.g., “Put on sunscreen today” or “Wow, it’s a hot one, you know what to do!”). The text message bank was developed by requesting suggestions for potential text messages from students, faculty and leadership at Operating Engineers Local 324. Since positive messages have been found to emphasize the good and appeal to the listener’s desire for good, happy emotions [45], which we want to convey during the Operating Engineers working hours, negatively oriented text messages were excluded from the message bank.

#### Education, sunscreen, and text message reminders

Just as multimodal interventions such as surgery and radiation can be used to treat skin cancer, multimodal behavioral interventions may reduce sun burning and prevent skin cancer. To determine if the combination of these interventional components results in improvements above and beyond the individual parts, both sunscreen and text messaging interventions will be provided in addition to education.

### Measures

#### Independent variables

The independent categorical variables will consist of 3 dummy variables created from the four sun treatment conditions using the education only as the reference group.

#### Dependent variables

Since self-report has been shown to be a valuable measure when skin reflectance measurement is not feasible [46], sun exposure will be assessed using two validated questions [47]. In the past summer, on the days when you were outside in the sunlight, how often did you use sun block (never, some of the time, about half the time, most of the time, always)? On average, how many times did you get a sunburn this past summer (0, 1, 2, 3, 4 or more times)?

#### Sun exposure covariates

To measure sun exposure, Operating Engineers will be asked four questions. About how many times in your life do you recall having had a sunburn severe enough to cause your skin to blister (write in number)? In general, during the summer week days, about how many hours a day are you outside between 10 am and 3 pm? In general, during the summer holidays and weekends, about how many hours a day are you outside between 10 am and 3 pm (less than an hour, 1 to 2 hours, 2 to 3 hours, 3 to 4 hours, 4–5 hours)? Which best describes how your skin generally reacts to the sun when you’re not using any sun protection (always burn-unable to tan, usually burn-then can tan if I work at it, sometimes mild burn-then tan easily, rarely burn-tan easily) [47]?

#### Health belief model covariates

The impact of the intervention on the four components of the Health Belief Model, which have been empirically supported by previous studies, will be measured with questions (rated on a 5-point scale) similar to those used in our tobacco cessation studies [48]. Perceived susceptibility will be measured by the questions: How likely do you think you are to sunburn next summer? and How likely do you think you are to develop skin cancer? Perceived severity will be measured by the questions: How bad would it be for you to get sunburned? and How bad would it be for you to get skin cancer? Perceived benefits to using sun protection will be measured by the question: How important is it that you prevent sun burning? and How important is it that you prevent skin cancer? Perceived barriers will be measured by the question: How difficult will it be to apply sun protection regularly [49,50]?

Several mediators of the Heath Promotion Model will also be measured. Self-efficacy will be measured by the question: How confident are you that you can apply sun protection regularly? Cues to action, which will vary depending on the intervention to which subjects were randomized, will be measured by the question: The educational presentation and/or mailed sunscreen and/or text message reminders increased the likelihood that I will use sunscreen.

Psychological status will be measured using the well validated Center for Epidemiologic Studies/Depressed Mood Scale (CES-D-SF) [51]. Since medical comorbidities can increase health motivation, comorbidities will be measured using a validated self-report instrument [52]. Since demographic factors may mediate sun protection behaviors, age, sex, ethnicity/race, educational level, marital, and veteran status will be asked.

#### Health behavior covariates

Since poor health habits have been shown to cluster together [53-55] and our prior research has shown that problem drinking, greater body mass index (BMI) and greater physical activity levels predict use of sunscreen and/or sun burning [23], questions will be asked about other health behaviors including smoking (Heavy Smoking Index) [56], problem drinking (Alcohol Use Disorders Identification Test-Hepatitis C: AUDIT-C) [57], diet (two questions on fruit and vegetable intake from the validated Willett food frequency questionnaire) [58], a validated physical activity questionnaire [59], and Medical Outcomes Study sleep quality survey [60]. Self-reported height and weight will be used to determine BMI (weight in kilograms divided by the square of height in meters).

#### Job characteristic covariates

To determine if job characteristics impact sunscreen use and sun burning, several questions will be asked. Which type of work do you do (check all that apply): commercial, residential, heavy/civil, and road? What type of cab does the equipment you usually operate in the summer months have: Completely enclosed (i.e., windows, roof, and door); Partially enclosed (i.e., roof but no windows or door); Completely open (i.e., no roof, windows, or door)? How often do you operate heavy equipment during the summer months with the doors and/or windows of the cab open (More than 75% of the time; 50-75% of the time; 25-49% of the time; Less than 25% of the time)? Regional differences in sunscreen use and sun burning will be explored by the question: I am most likely to work in lower Michigan (below Saginaw, Midland and Muskegon), upper Michigan (above Saginaw, Midland and Muskegon, but not Upper Peninsula), Upper Peninsula, other (write in). Questions (yes/no) will be asked on whether they use other protective equipment including respirators, steel toed boots, high-visibility clothing, fall protection, hearing protection, safety glasses, hard hats, and work gloves. Standard industry (yes/no) questions will be asked on occupational exposures including asphalt fumes, heat stress, concrete dust, welding fumes, lead, benzene, asbestos, solvents, and silica [61].

#### Additional evaluation questions

Evaluation questions (rated on a scale of strongly disagree to strongly agree) will vary depending on the intervention subjects received and will include: The educational presentation was easy to understand. The educational presentation and or mailed sunscreen and/or text message reminders were helpful. Overall I was satisfied with the presentation and or mailed sunscreen and/or text message reminders. I would recommend the sun protection intervention to others. A write in question will ask: What, if anything, would you change about the sun protection intervention?

### Data analysis

Descriptive statistics will be computed for all variables at all measurement times. Distributions will be examined and variables will be transformed to normality if needed. The equivalence of the groups on pretest data will be tested using χ^2^ tests of association for categorical variables and Analysis of Variance (ANOVA) for continuous variables. Then analyses to meet the aim will be conducted by paired t-tests, RM-ANOVA, t-tests comparing change scores and by linear regression analyses. If the groups being compared in the RM-ANOVAs differ on the covariates, the analysis will be changed to repeated measures analysis of covariance (RM-ANCOVA) with the differing covariates controlled. Multiple imputation will be used to replace the values of missing data. An intent-to-treat approach will be used so that subjects are considered to be in the condition to which they were randomized despite how much they actually used that type of care. Analyses will be conducted by two-tailed tests with alpha of .05.

## Discussion

The study design is novel in that no studies have compared the individual efficacy of four interventions varying in intensity as well as the average effects of the four interventions. Moreover, no studies have tested the efficacy of mailed sunscreen, which is expected to reduce barriers to use similar to how mailed nicotine replacement reduces barriers to use and enhances smoking quit rates [62]. By incorporating the educational component into regularly scheduled safety trainings that Operating Engineers are already attending for their job, it is expected that participation rates will be high. The setting is novel in that work site interventions have not been tested among Operating Engineers who have high sun burning rates. This proposal is timely in that the new federal Affordable Care Act (ACA) [63] contains numerous provisions to encourage prevention including worksite initiatives as most adults spend almost one-third of their time in the workplace.

Taking the best interventions from this trial, we have the possibility of developing a wider scale trial in conjunction with the International Training Center, which services North America (including the United States, Mexico, and Canada). The intervention may be even more beneficial to southern states with hotter climates than Michigan. Wide scale dissemination of an efficacious sun protection intervention has the potential to decrease sun burning and skin cancer rates among this population.

## Consent

Written informed consent was obtained from the participants for publication of this report.

## Abbreviations

RM-ANOVA: Repeated measures analysis of variance; UV: Ultra violet; RCT: Randomized controlled trial; USFDA: U.S. Food and Drug Administration; USPSTF: U.S. Preventive Services Task Force; AAD: American Academy of Dermatology; SPF: Sun protection factor; CES-D-SF: Center for Epidemiologic Studies/Depressed Mood Scale; BMI: Body mass index; AUDIT-C: Alcohol Use Disorders Identification Test-Hepatitis C; ANOVA: Analysis of variance; RM-ANCOVA: Repeated measures analysis of covariance; ACA: Affordable Care Act

## Competing interests

The authors declare that they have no competing interests.

## Authors’ contributions

SD, as a Principal Investigator, conceived of the study, and participated in its design and coordination and helped with drafting the manuscript. DR participated in the design of the study and performed the statistical analysis. AW helped with drafting the manuscript and coordination of the study. SHC participated in writing the manuscript. All authors read and approved the final manuscript.

## Pre-publication history

The pre-publication history for this paper can be accessed here:

http://www.biomedcentral.com/1471-2458/13/273/prepub
